# Primary Prevention of Airway Allergy

**DOI:** 10.1007/s40521-018-0190-4

**Published:** 2018-11-05

**Authors:** Johanna Wikstén, Sanna Toppila-Salmi, Mika Mäkelä

**Affiliations:** 1 Skin and Allergy Hospital, Helsinki University Central Hospital, Meilahdentie 2, 00250 Helsinki, Finland; 2 Haartman Institute, Medicum, University of Helsinki, Helsinki, Finland

**Keywords:** Asthma, Allergic rhinitis, Smoking, Pregnancy

## Abstract

**Purpose of review:**

The aim of this paper is to review and summarize the current knowledge of prevention of airway allergy.

**Recent findings:**

Allergic rhinitis and asthma are allergic airway diseases. Due to their increasing incidence and socioeconomic burden, allergic airway diseases have recently gained attention worldwide. The primary prevention of allergic airway diseases focuses on offspring’s gestational and childhood environment, such as maternal smoking and diet during pregnancy and breastfeeding as well as exposure to environmental microbes and irritants.

**Summary:**

Asthma and allergic rhinitis are a major public health problem worldwide. They have increasing prevalence and thus attempts to their prevention are mandatory. Rapid action needs to be taken to restrain smoking among children and adolescents in order to prevent burden of allergic airway diseases. Exposure to pollution and environmental issues concerning hygiene and lifestyle would also need to be actively addressed. More evidence is still needed in order to draw linings concerning maternal diet and other factors during the offspring’s whole life span.

## Introduction

Allergic rhinitis (AR) and atopic asthma are chronic inflammatory allergic airway diseases. AR is caused by allergen binding to specific IgE in allergen-sensitized subjects leading to rhinorrhea, obstruction, itch, sneeze, and fatigue symptoms [1]. AR is connected to allergic and non-allergic co-morbidities such as asthma, allergic conjunctivitis, atopic dermatitis, and chronic rhinosinusitis (CRS). Asthma is characterized by chronic inflammation with mucus hypersecretion, edema, variable obstruction, and fatigue. Asthma is not a single disease entity; it encompasses different phenotypes, both in children and adults, and the most commonly described phenotypes include allergic and non-allergic asthma [2]. Childhood asthma is characterized by a predominance of allergic multi-morbidity in males [3]. In adults, a large variety of allergic and non-allergic asthma phenotypes exist with a female predominance. Asthma can roughly be divided into Th1- and Th2-high phenotypes. The latter includes allergic and/or eosinophilic disorders varying from mild to severe progressive forms. Severe eosinophilic forms, such as acetylsalicylic acid (ASA-) exacerbated respiratory disease (AERD), are more common in adults. Few approaches studied so far have shown encouraging signals in prevention of asthma. Part of the problem and also the solution may lie in over-simplification of terminology. Asthma is a complex inflammatory disease which cannot be considered a single entity. It is rather a heterogenous dynamic immunological disorder strongly influenced by gene/environment interactions.

AR and asthma are major public health problems worldwide, with over 300 million people worldwide affected [4, 5]. The prevalence of AR varies according to previous studies between 15 and 50% of the population [6, 7]. A recent Swedish study showed the prevalence of AR at the age of 12 to be 13% [8]. Taking also in to account that mild symptoms do not require medical treatment and the fact that most of the patients outgrow their allergy, especially concerning food allergy, the precise prevalence and therefore socioeconomic impact is difficult to calculate. There is large variability of particularly childhood asthma prevalence and incidence in different parts of the world. After many decades of ever increasing asthma rates in the Western world, we seem to have recently reached a plateau and in some places even a decrease at least in incidence from the beginning of 2000 in many developed countries. Children migrating from low-income areas to higher socioeconomic areas have a lower prevalence of asthma suggesting a critical time window for childhood early in life. This implies to a possibility to prevent asthma since there are obviously biological predisposing factors influenced by the environment. On the other hand, there are biological borders, likely genetic in nature, limiting the number of asthmatics in the whole population. It should be kept in mind that up to 85% of asthma patients have AR, and on the other hand, 15–38% of AR patients have asthma [9].

There are five problems throughout the published studies that are (1) small children have during respiratory infections respiratory noises and parents label them often as “wheeze” to please physicians’ obsession. However, more than 30% of parents use other words and 30% label other sounds for wheeze; (2) typically viral wheezers end up getting control medication in most countries and they are often maintained on this until school-age, despite symptoms resolving with age. These children are counted as asthmatics since they have “doctor diagnosed asthma”; (3) most studies do not try to separate those with early allergy and asthmatic symptoms from viral wheezers; (4) many of the wheezers with more permanent phenotype do not get the proper label of asthma; and (5) studies rarely involve objective measurements.

Both atopic asthma and AR increase in population. Megatrends such as climate change, population growth, and urbanization might impact this increase [10] suggesting strong environmental impact on disease development in addition to its genetic predisposition. Prevention of allergy is recommendable, since its socioeconomic burden is heavy. The aim of this review is to gather the current knowledge of primary prevention of allergic airway diseases.

## Smoking and allergic airway diseases

Exposure to passive tobacco smoke as well as active smoking among adolescents and even children is amazingly high worldwide [11]. Smoking during pregnancy is common, and estimated rates vary from 17 to 30% [12]. Cigarette smoking is the single largest modifiable risk for all pregnancy-related morbidity and mortality [13, 14].

Numerous studies have confirmed the effect of external tobacco smoke on the risk of asthma and also on the severity of bronchial inflammation [15]. The most recent data indicate that this risk may be even transmitted by possible epigenetic mechanisms over the generation from grandmothers to grandchildren [16]. A recent meta-analysis found that on adults, active smoking did not increase risk of AR, but increased the risk for rhinitis. On the other hand, AR was associated with passive smoking in adults. In children and adolescents, both active and passive smoking increased the incidence of AR. The study group estimated that 14% of the AR is due to active smoking, therefore eliminating smoking among young and children, every seventh case of AR could be prevented [17]. Reduction of smoking, therefore, remains the easiest and one of the most concrete ways of practical asthma prevention [18].

Studies have shown that maternal smoking during pregnancy increases the risk for wheezing in childhood or asthma among offspring during early childhood [19], in preschool-age children [20], in adolescents [21], and in adults [22]. These findings highlight the need for strategies to encourage women of childbearing age and parents having children to permanently cease smoking in order to decrease AR and asthma risk of offspring.

## Allergen avoidance as prevention of airway allergy

It is not clear whether airway allergy can be prevented by allergen avoidance in families having risk for allergic diseases. Most studies aiming to reduce risk of asthma or allergy through controlling environmental exposure to allergens are inconsistent and have failed. The Isle of Wight study examined the effect of diets and extensive measures to reduce exposure to house dust mite (HDM). This study with a relatively small number of children (*n* = 120) considered at high risk for allergic disorders is the only trial which has shown reduction of mite sensitization and asthma persisting until the age of 18 years [23]. The much larger and more comprehensive Manchester study reported an opposite effect on mite sensitization [24]. A randomized controlled trial was performed in 2006, which found no decrease in asthma development comparing HDM avoiding families and those without [25]. Another study strengthened this theory by showing no differences in sensitization or wheezing periods in children either avoiding the allergen or not, according to dust samples [26]. An impaired lung function was shown to develop on children with high exposure to indoor allergens early in life compared to children without sensitization. On the other hand, high amount of viral upper respiratory tract infections in the first years of life may reduce the onset of asthma later in life [27].

At the moment, we do not fully understand the interaction between allergic sensitization and disease, not to talk about the clinical relevance of allergen exposure to development of asthma. It is clear that if an anaphylaxis has happened, then avoidance is recommendable. Also, in secondary prevention, avoidance seems to have a role [28].

## Inhaled indoor factors

HDM has been the most studied of indoor air particles in association with AR prevention. Especially in the tropical countries, house dust mite allergy rate has risen [29]. HDM allergy seems to be the most common cause for perennial AR and bedroom environment is gaining more interest, as avoidance of the contacts in sensitized individuals may lead to symptom relief. In the countries with exposure to different allergens due to seasonal changes makes the diagnostics more complex. A Cochrane review was conducted in 2010, in which seven reports concluded that HDM load can be reduced with combination of acaricides and bedroom environmental control programs. Reduction of AR symptoms is poorly assessed in these reports, and more specific studies are in request [30, 31••]. Also, other factors as excessive moisture and volatile organic compounds may play a role in airway allergy, but taken together, controversial knowledge exists of the effect of indoor particles to development of AR or asthma.

## Outdoor environmental factors

Climate change and therefore air pollution is thought to be one of the main reasons for increase of allergic diseases [10]. Especially, sulfur dioxide (SO_2_) is shown to increase AR. Increase in the levels of SO_2_ correlated in the doctoral consultations concerning AR [32]. In a cross-sectional questionnaire study, the authors concluded that increase in the levels of SO_2_ increased the prevalence in AR in Taiwanese school children [33]. Also, exposure to traffic-related pollutant during pregnancy is shown to increase the incidence of AR, as well as asthma and eczema [34]. Exposure to extreme heat has speculated to increase the incidence of hay fever [35]. Taken together, evidence exists that outdoor air pollution is associated with airway allergy.

Several studies reported nearly 20 years ago that children grown up in farm environment develop less asthma and allergies. This effect has been studied extensively and it is now contributed to contact with farm animals and their microbes. The most recent studies from Amish and Hutterite populations demonstrated that Amish children have much lower prevalence of asthma than Hutterite children, despite the similarities in ancestry and most lifestyle factors associated with asthma risk [36]. At the moment, the data suggest that airborne substances likely derived from animals and their microbes shape innate immune pathways to finally produce protection from asthma. Several trials are now underway to examine whether for example bacterial lysates could prevent development of asthma.

## Probiotics, vitamin D, and obesity

During the last few decades, interest towards probiotics has risen. Several trials have been able to demonstrate a clear decrease in risk of atopic eczema, when both pregnant mothers and newborns have been given supplementation of probiotic bacteria such as Bifidobacterium lactis or Lactobacillus rhamnosus. To date, none of these trials have shown any sign of asthma prevention. It seems that even atopic eczema dissociates from atopic sensitization. The central biological and immunological mechanisms in development of asthma or eczema may be distant [37]. A large (*n* = 4783) questionnaire study from Poland confirmed that probiotics would not have any preventive effects on AR when exposure is done in early childhood. In contrast, adolescents may benefit from probiotics in prevention of allergic diseases, but their role remains unclear [38].

In a recent systematic review, the role of vitamin D in primary prevention of allergic diseases was assessed in four populations: pregnant and breastfeeding mothers, infants, and older children. No clear association between D vitamin and prevention of airway allergy was found [39, 40]. There is lacking knowledge of the association between obesity and AR. In a Chinese survey study (*n* = 4132), the prevalence of allergic rhinitis and atopic dermatitis was higher among obese children compared to children with normal weight [41].

## Perinatal dietary factors

During the breastfeeding-period and first months of life, nutrition of the baby is regarded as a major factor in development of allergic diseases. Diet of pregnant and lactating mothers as well as food introduction age are the major focuses of the studies. There is not enough evidence on AR with dietary consumption of the mom during the pregnancy or the breastfeeding period. It is shown that if milking is not sufficient, highly hydrolyzed formulas of cow milk decrease the incidence of atopic eczema, but not asthma or AR [42••]. Polyunsaturated fatty acids (omega-3- and 6) were tested in a randomized controlled trial in Australia and no significant difference on allergic disease onset was found between the study groups [25]. Numerous follow-up cohorts have addressed the significance of breastfeeding on development of asthma. The results have been often inconsistent and the studies are often confounded by selection bias and reverse causality. Even several meta-analysis studies done over the years have not been able to solve the dilemmas and do not demonstrate a consistent protection from asthma. It is, however, obvious that breastfeeding should be recommended in all guidelines for many other health benefits [43].

## Maternal nutrition

The nutrition of mothers during pregnancy: dietary patterns and selective supplementation as well as levels of iron, vitamin D, folic acid, and other nutrients may have protective as well as adverse effects on the evolution of atopic diseases in the offspring [44]. However, all the evidence is at the moment circumstantial and today only one published trial on nutritional intervention of mothers has been able to show a clear decrease in asthma risk. In a Danish study, pregnant mothers received fish oil or placebo and their offsprings were followed for 3 years. The risk of the children’s persistent wheeze and infections of the lower respiratory tract infections was reduced by approximately one third [45].

## Secondary prevention with immunotherapy

Only few studies have addressed the possibility to prevent asthma in those with AR. The GAP trial is the only large, randomized, placebo-controlled study on this subject. The investigators recruited 812 children between 5 and 12 years of age with a grass pollen allergic rhinitis to a sublingual immunotherapy study which comprised 3 years of treatment and 2 years of follow-up [46]. Although the IT tablet reduced the risk for asthma symptoms and medication, there was no difference in time to onset of asthma, leaving still the question open whether we truly can prevent this complex multifactorial disease by immunotherapy against one allergen. One has to note that none of the studies with allergen-specific immunotherapy have been able to show a change in the general immunity from Th2 high to Th2 low, but the effect is always truly and only allergen-specific.

## Could asthma be prevented by preventing atopy?

Atopy is an important risk factor of asthma development and family history of atopy is highly significant for development of atopy. It is, therefore, logical to think that prevention of atopy would be of uttermost importance to prevent asthma. However, we still understand poorly why up to 75% of teenage asthmatics are atopic but much smaller degree of atopics are asthmatic [47, 48].

In the early days of atopy and asthma research, it was presumed that most children would follow a pattern called “atopic march,” in other words, asthma would succeed rhinitis which again is preceded by eczema and food allergy. In point prevalence, this concept looks valid. Also, clinicians see small children often presenting with eczema and food allergy and older children seem to acquire allergic asthma. However, in the view of modern cohort studies, this is not the case in the majority of asthmatic children. The most carefully done study examining this was done in UK where two birth cohorts were combined producing a massive amount of data from more than 10.000 children. In this study employing complex mathematical models, only 6% of the children with any atopic disease followed the classical atopic march [49]. Moreover, up to 70% of children with eczema do not progress to airway allergy.

It has been speculated if continuous washing of skin with soap brakes the skin barrier by making the skin too dry and it has been suggested that if in the early childhood, the skin was washed with creams instead of soap, the way of the whole allergic march could be changed [50•].

## Conclusions

Current knowledge of primary prevention of allergic airway diseases is limited, except for smoking cessation. Childhood AR and asthma risk are increased by exposure to smoking. Hence, asthma and AR offspring could be reduced and airway health could be improved by encouraging parents to permanently cease smoking. There is limited or controversial knowledge of other environmental factors and their effect on airway allergies.

## Future needs

Western urbanization and lifestyle changes are claimed to be the main reason for the increase of airway allergy [51–54]. There is a global need of prevention of the current epidemics of chronic allergic respiratory diseases. Studies on prediction and prevention of allergic diseases are needed. Programs of better education and successes of children and adults in the case of the prevalence and burden of allergic airway diseases are needed, such as allergy programs [55]. This would increase airway health and also general health and wellbeing.

## Abbreviations

AR: Allergic rhinitis
CRS: Chronic rhinosinusitis
ASA: Acetylsalicylic acid
AERD: Exacerbated respiratory disease
HDM: House dust mite
SO_2_: Sulfur dioxide
